# Impact of uteroplacental insufficiency on postnatal rat male gonad

**DOI:** 10.1530/JOE-16-0418

**Published:** 2016-12-13

**Authors:** Valentina Pampanini, Daniela Germani, Antonella Puglianiello, Jan-Bernd Stukenborg, Ahmed Reda, Iuliia Savchuk, Kristín Rós Kjartansdóttir, Stefano Cianfarani, Olle Söder

**Affiliations:** 1 Department of Women’s and Children’s HealthPediatric Endocrinology Unit, Q2:08, Karolinska Institutet and University Hospital, Stockholm, Sweden; 2 Department of Systems MedicineTor Vergata University, Rome, Italy; 3 Dipartimento Pediatrico Universitario Ospedaliero ‘Bambino Gesù’ Children’s Hospital – Tor Vergata UniversityRome, Italy

**Keywords:** intrauterine growth restriction, developmental origins of health and disease, uteroplacental insufficiency, testis

## Abstract

Prenatal events such as intrauterine growth restriction can affect gonadal development of the offspring and have an impact on reproductive health. To investigate the effects of intrauterine growth restriction induced by uterine artery ligation on the postnatal rat testis. Pregnant rats underwent uterine artery ligation at day 19 of gestation. Offspring were killed at 5, 20 and 40 days *post-partum* (d*pp*). At killing, one gonad was snap-frozen in liquid nitrogen and processed for RNA and steroid extraction. The other gonad was formalin-fixed for histology. Gene expression was analyzed by TaqMan Low-Density Array. Intratesticular testosterone, estradiol and serum gonadotrophins were measured. Thirty genes were dysregulated in intrauterine growth-restricted rats compared to controls, among which markers of Sertoli cell and Leydig cell function, cell metabolism and growth factors. Testis weights were significantly reduced at 5 and 20 d*pp* in intrauterine growth-restricted rats and caught-up by 40 d*pp*. Accordingly, Sertoli cell number was significantly lower in 5 d*pp* intrauterine growth-restricted rats. At 20 d*pp*, intratesticular testosterone was significantly increased in intrauterine growth-restricted rats, whereas serum gonadotrophins were unchanged. IUGR altered the gene expression in the rat testes up to peripubertal age and reduced testis size and Sertoli cell number in neonatal age. Multiple mechanisms encompassing genetic changes and steroid production by the testis may be involved in the catch-up growth phase that restored testis size by 40 d*pp*. Permanent consequences on organ function and gamete integrity cannot be excluded and deserve further investigations.

## Introduction

Adverse conditions during fetal life, such as low nutrient and/or oxygen supply from the placenta, can lead to intrauterine growth restriction (IUGR) and low birth weight. Besides affecting body growth, a suboptimal intrauterine environment can influence the expression of fetal genes and has an impact on growth and development of multiple organs and long-term health. This principle, pioneered by David Barker and coworkers in the early 90s, has later been termed ‘Developmental Origins of Health and Disease’ (DOHaD) hypothesis and is nowadays supported by broad scientific evidence (Barker 1990, Hales & Barker 2001, Silveira *et al*. 2007).

Intrauterine stress produces fetal gene reprogramming generating oxidative stress and the production of reactive oxygen species (ROS). Those molecules can directly bind to specific sites on DNA and cause genetic and epigenetic modifications, e.g. by interfering with DNA methylation and histone configuration (Thompson & Al-Hasan 2012).

IUGR and low birth weight are associated with an increased risk of cardiovascular and metabolic diseases in adult life (Barker *et al*. 1990, 1993, Hales *et al*. 1991, Law *et al*. 1992, Barker 1995, Forsen *et al*. 1999, Arends *et al*. 2005). As indicated by experimental IUGR models, permanent modification in the genetic programming can account for this association. Fetal growth restriction dysregulates key enzymes involved in metabolic processes, especially those related to glucose metabolism (e.g. phosphoenolpyruvate carboxykinase (*Pepck*), glucose transporter 1 and 4 (*Glut-1* and *Glut-4*)), and these changes have been identified in multiple organs, such as skeletal muscle, liver and heart (Tsirka *et al*. 2001, Peterside *et al*. 2003, Selak *et al*. 2003, Vuguin *et al*. 2004).

In addition to the well-known association between low birth weight and cardiometabolic diseases, IUGR has been related to an increased risk of male reproductive health disorders, such as cryptorchidism, hypospadias and testicular cancer, grouped under the definition of testicular dysgenesis syndrome (TDS) (Skakkebaek *et al*. 2001, Main *et al*. 2006). Previous studies reported alterations of puberty and hormonal levels in boys, but the results are conflicting and difficult to compare due to different inclusion criteria and definitions used (Persson *et al*. 1999, Hokken-Koelega 2002, Veening *et al*. 2004, Jensen *et al*. 2007, Hernandez & Mericq 2008, Dupont *et al*. 2012, Zambrano *et al*. 2014).

In rats and sheep, gonadal morphology, functionality and sperm parameters are altered by modifications of the intrauterine environment during critical windows of fetal development, but most of these experimental studies have used maternal nutrition manipulation to produce IUGR animals (Engelbregt *et al*. 2000, 2002, Rae *et al*. 2002, Zambrano *et al*. 2005, Kotsampasi *et al*. 2009, Genovese *et al*. 2010, Toledo *et al*. 2011).

In Western industrialized countries, uteroplacental insufficiency (UPI) is the most common cause of IUGR (Gagnon 2003). Therefore, animal models using surgically induced UPI are more relevant to pregnancies in developed countries than manipulation of maternal diet (Vuguin 2007).

To our knowledge, only two studies have investigated the effects of IUGR induced by UPI on the testis. UPI coupled with postnatal undernutrition delayed the onset of puberty and altered testicular function by a reduction of testosterone levels (Engelbregt *et al*. 2000, 2002).

Here, we used a model of UPI initiated at day 19 of gestation in pregnant rats, to investigate the effect of IUGR on the gene expression, structure and function in the postnatal testis.

## Material and methods

### Animals

All protocols for the study were approved by the Committee for Animal Research of Tor Vergata University, Rome, Italy. Animal experiments were performed according to the Guide for the Care and Use of Laboratory Animals of the National Institutes of Health (NIH Publication No. 85-23, revised 1996). All procedures complied with Italian regulations for laboratory animal care, according to the guidelines and under supervision of the Animal Technology Station, Interdepartmental Service Center, Tor Vergata University, Rome, Italy.

The animal model was developed as previously described (Puglianiello *et al*. 2007). Time-dated Sprague–Dawley pregnant rats (Charles River Laboratories Inc.) were delivered to our animal facilities at least three days prior to the first surgery (day 15–16 after conception). The supplier checked the vaginal plug to confirm mating and defined day one of pregnancy as the day immediately following the night where the animals mated. After arrival at our facility, animals were allowed standard chow diet and water *ad libitum* and maintained on a 12 h light/darkness cycle at 21°C. IUGR was induced by bilateral ligation of the uterine arteries on day 19 of gestation according to a modification of the method of Wigglesworth (1964). Animals were anesthetized with intramuscular injections of xylazine (10 mg/kg) and ketamine (50 mg/kg) (Sigma-Aldrich), a four to five cm long lower midline abdominal incision was made and the uterine arteries of both sides were localized and ligated. Suture was placed around both the uterine arteries and then either tied or withdrawn before closing the abdomen. The latter protocol (*sham* procedure) was used to generate control animals, aiming at the limitation of confounding factors, such as anesthesia and surgical stress. The surgical procedure lasted 10–15 min, and animals recovered within three-four hours from the administration of anesthetic drugs. Dams were returned to the animal facilities and housed in individual cages. Dams delivered spontaneously during the night between day 22 and 23. Pups were weighed in the morning of day 23. Significant IUGR was considered as a birth weight <−2 SDS compared to the mean birth weight of the control litter. After birth, dams were killed and pups were assigned to feeding dams. To standardize postnatal conditions (i.e. to ensure an equal access to lactation) litter sizes were reduced to six offspring. Pup weights were recorded on day 0 and subsequently once a week until the end of experiments. At 21 days *post-partum* (d*pp*), pups were weaned onto standard rat chow diet (Mucedola S.R.L., Milano, Italy), separated from dams and placed in groups of three to five, with the males and females separated. For the purpose of this study, only male pups were selected. Animals were killed through cervical dislocation at postnatal day 5, 20 and 40 d*pp*. To limit confounding factors and to ensure biological variability of the observed phenotype, each group of IUGR and *shams* at 20 and 40 d*pp* included six animals from at least three different litters. For the 5 d*pp* old animals, comparisons were made between seven IUGR and four shams, respecting the same principle of litter variability.

### Testicular histology and immunohistochemistry

At killing, testes were immediately excised from killed animals, trimmed of fat and connective tissue and weighed. For histological purpose, one gonad was fixed in 4% paraformaldehyde (PFA; P/N15812-7, Sigma-Aldrich) overnight at 4°C, followed by serial dehydration in 30, 50, 70 and 80% aqueous ethanol for 24 h at each concentra­tion, at room temperature (RT). Afterward, samples were placed for six hours in 96% ethanol, and then in 99.6% ethanol and 100% butyl acetate (P/N 45860, Sigma-Aldrich), each overnight at RT and finally embedded in paraffin (Paraplast X-TRA; P/N P3808, Sigma-Aldrich) at 61°C overnight. Paraffin-embedded tissue was cut to a thickness of five micrometer, using a Biocut sectioning machine (Reichert-Jung, NY, USA), mounted on microscope slides (P/N10143352, Superfrost Plus, Thermo Scientific) and placed at 37°C overnight. Tissue sections were dewaxed with xylene (P/N 02080, HistoLab, Gothenburg, Sweden) for 10 min and then serially rehydrated with 99.6, 96 and 70% aqueous ethanol, each step being performed twice for five minutes.

For histological evaluations, samples were subsequently stained with periodic acid-Schiff (PAS) kit (P/N 395B-1KT, Sigma-Aldrich). In brief, after washing twice with distilled water, samples were incubated for five minutes with periodic acid and then rinsed with tap water followed by distilled water for two times. Samples were then incubated for 15 min with Schiff’s reagent and washed again as previously described. Slides were finally incubated with hematoxylin solution modified according to Gill III for two min, washed with tap water for three minutes, dehydrated with increasing aqueous ethanol solutions and 100% xylene and finally mounted with Entellan new (P/N 1079610100, Merck) and cover glass.

After dewaxing in xylene and partial rehydration in two 100% ethanol baths, samples for immunohisto­chemical (IHC) staining were incubated with 3% H_2_O_2_ in 96% ethanol for 10 min at RT for endogenous peroxidase blocking. Afterwards, tissue sections were subjected to antigen retrieval using citrate buffer (pH 6.0) at 95°C for 30 min, cooled down for 20 min and then incubated with 10% goat serum in phosphate-buffered saline (PBS) plus 1% bovine serum albumin (BSA) for 30 min at RT. Slides were subsequently incubated with primary antibody against Wilms tumor-1 (WT1) (P/N ab89901, Abcam) or unspecific IgGs (for negative control) dissolved in 10% goat serum, 1% BSA-1 × PBS, overnight at 4°C. After washing three times for five minutes each with 1× Tris-buffered saline (TBS) plus 0.01% Tween20 (P/N P1379, Sigma-Aldrich), slides were incubated with biotinylated secondary antibody (P/N ab64256, Abcam), and then with avidin–biotin–peroxidase complex prepared using Vectastain ABC kit (P/N PK-6100, Vector Laboratories, Burlingame, CA, USA) for 30 min at 37°C. After washing again three times, slides were stained with DAB (P/N SK-4105, Vector Laboratories) for 30–60 s at RT, washed twice for five min in H_2_O, counter-stained with Hämalaun solution (1.09249.1000, Merck), rinsed for five min with running tap water, dehydrated with gradually increasing concentrations of ethanol, cleared with xylene and mounted with cover glass.

### RNA isolation and cDNA synthesis

Total RNA was extracted from whole frozen testes and stored at −80°C using an RNeasy Mini Kit (P/N 74104, Qiagen). In brief, samples were dissolved in Qiagen lysis buffer and then homogenized twice for 30 s in an ULTRA-TURRAX T25 homogenizer (JANKE and KUNKEL, Staufen, Germany). Subsequently, the supernatant was mixed with 0.5 times its volume of 70% ethanol. The mixture was then transferred to RNeasy spin columns and processed according to the manufacturer protocol.

RNA concentrations were quantified using a single-beam UV/vis spectrophotometer (P/N 6132000032, Eppendorf, Hamburg, Germany). To generate cDNA, one microgram of total RNA was reverse-transcribed on a thermocycler (P/N 4359659, Applied Biosystems) using random hexamers in a total reaction volume of 20 µL. The IScript cDNA synthesis kit (P/N 170-8891, Bio-Rad) was used as instructed by the manufacturer.

### TaqMan low-density arrays (TLDAs)

TLDA cards (P/N 4342259, ABI, Hilden, Germany) were used for comparative analysis of gene expression of IUGR and control animals, according to the manufacturer’s protocol. Briefly, the TLDA cards are based on TaqMan chemistry where gene expression of a panel of 96 genes is analyzed in one run. The TLDA cards were pre-loaded with 96 TaqMan gene expression assays of importance for proliferation, apoptosis, cellular energy, germ cell (GC) and somatic cell function and differentiation, and six endogenous controls were assigned for normalization. Gene expression was normalized to the mean of five out of six endogenous controls (Actin beta, Beta-2-microglobulin, Catenin beta 1, Eukaryotic translation elongation factor 1 alpha 1 and Glyceraldehyde-3-phosphate dehydrogenase) of the same sample (d*C*_T_), selected according to their stability. Data from one randomly chosen animal were used as a calibrator (dd*C*_T_) and data from all other animals were normalized to it. Gene expression was finally presented as relative expression (using the fold change (2^−ddCT^) method for calculation).

TaqMan gene expression Master Mix (P/N 4369510, Applied Biosystems) was used when running the TLDA assays.

### Intratesticular steroids measurement

Intratesticular testosterone and estradiol were assessed as a measure of Leydig cells (LC) and Sertoli cell (SC) enzymatic activity in 20 and 40 d*pp* animals. Steroids were extracted from samples stored at −80°C by adding 0.5 mL ethyl acetate (P/N 1096232500, Merck), followed by vigorous automated shaking for 15 min. After centrifugation for two min at 16,000*** g***, the resulting supernatant was separated and the residue was re-subjected to the same procedure. The two ethyl acetate extracts were combined and evaporated overnight; the pellet obtained was dissolved in PBS. The COAT-A-COUNT RIA kit (P/N TKTT2, Siemens) and DRG Estradiol ELISA (P/N EIA-2693, DRG International, Inc., Springfield Township, NJ, USA) were used to quantify testosterone and estradiol, respectively, with intra-assay variability CV of less than 5% and inter-assay variability CV of less than 10% for both, according to the manufacturer’s protocol. Each sample was analyzed in duplicate, and values were extrapolated from a standard curve prepared with known concentrations of testosterone and estradiol provided with the kits.

### Serum gonadotropins measurement

Luteinizing hormone (LH) and follicle-stimulating hormone (FSH) serum levels were measured in 20 and 40 d*pp* animals by ELISA kit (P/N CEA441Ra and CEA830Ra, respectively; USCN Life Science, Inc. Houston, TX, USA), with intra-assay variability CV of less than 10% and inter-assay variability CV of less than 12% for both in accordance with manufacturer’s protocol.

### Statistical analysis

To detect a difference in average *z*-scores between the control and treated of 0.5 with 90% power at the 5% significance level, six animals per group were included in the study, except for the 5 d*pp* group that was composed by 7 IUGR and 4 *sham* animals. Data were expressed as mean ± s.d. Statistical analyses were performed using Student’s *t*-test to compare the IUGR group with the controls. Differences were considered statistically significant at *P* ≤ 0.05.

## Results

### IUGR-affected network of selected genes

**Figure 1:**
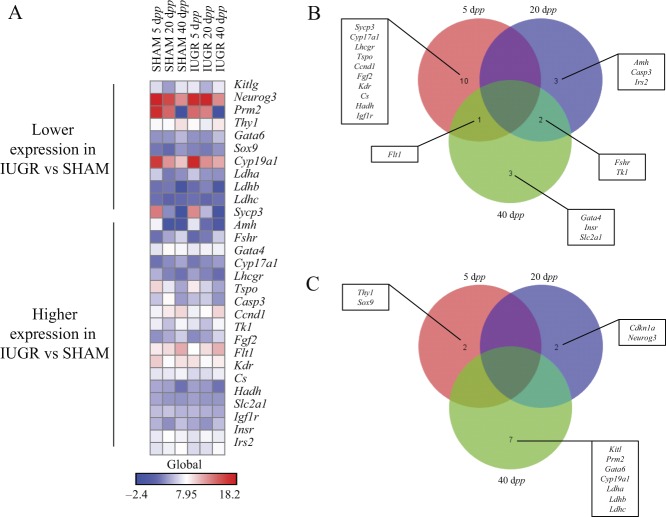
TaqMan Low-Density Array analysis of gene expression in IUGR and *sham* animals at 5, 20 and 40 d*pp*. (A) Significantly lower and higher expression (*P* < 0.05) of genes in IUGR animals relative to *sham* ones. (B) Specific differences in gene expression between animal groups, with higher expression in IUGR vs *sham* animals, and (C) lower expression in IUGR vs *sham* animals. For gene abbreviations and corresponding names see Supplementary Table 1.

We analyzed the relative quantitation of gene expression of 90 different markers of specific cellular functions in the rat testis at 5, 20 and 40 d*pp* (Supplementary Table 1, see section on supplementary data given at the end of this article) by TaqMan Low-Density Array (TLDA). A total of 30 genes were differentially expressed in the testes of IUGR vs sham rats (Supplementary Table 2). Among those, 19 genes showed significantly higher expression and 11 genes showed significantly lower expression in IUGR than in *sham* animals (Fig. 1).

Genes involved in the regulation of cellular energetic homeostasis were modulated in IUGR animals. Expression of citrate synthase (*Cs*) and hydroxyacyl-CoA dehydrogenase (*Hadh*) was upregulated in IUGR rats at 5 d*pp*, compared to controls (*P* < 0.02), whereas at 40 d*pp*, lactate dehydrogenase-a, -b and -c (*Ldh-a*, *Ldh-b* and *Ldh-c*) were downregulated (*P* < 0.002, *P* < 0.02, *P* < 0.02, respectively) and solute carrier family 2 member 1 (*Slc2a1*) was upregulated (*P* < 0.01) in IUGR rats compared to controls.

Three genes controlling cell proliferation, cyclin D1 (*Ccdn1*), cyclin-dependent kinase inhibitor 1A (*Cdkn1a*) and thymidine kinase 1 (*Tk1*) showed a significant congruent variation at the three age points (5, 20 and 40 d*pp*, respectively).

Several genes marking SC and LC function were affected by IUGR. Expression of the SC marker SRY (sex-determining region Y)-box 9 (*Sox9*) was decreased in IUGR rats at 5 d*pp*, compared to *shams* (*P* < 0.02). At 20 and 40 d*pp*, follicle-stimulating hormone receptor (*Fshr*) was significantly upregulated in IUGR rats (*P* < 0.05 and *P* < 0.02, respectively), whereas Kit ligand (*Kitl*) was downregulated in IUGR rats 40 d*pp*, compared to *shams* (*P* < 0.02).

Moreover, at 5 d*pp*, two genes involved in steroidogenic pathways for androgen synthesis in LC, cytochrome P450 family 17 (*Cyp17*) and translocator protein (*Tspo*), were both upregulated in IUGR rats (*P* < 0.05 and *P* < 0.04, respectively). At 20 d*pp*, no variations were detected, whereas at 40 d*pp*, cytochrome P450 family 19 (*Cyp19*) expression was downregulated in IUGR rats (*P* < 0.01).

Given the known role of insulin and insulin-like growth factors (IGFs) in the regulation of somatic cells development and function within the testis, testosterone production and spermatogenesis itself (Gnessi *et al*. 1997, Sofikitis *et al*. 2008, Pitetti *et al*. 2013), we investigated the expression of *Igf1*, Igf1 receptor (*Igf1r*), Igf-binding protein 3 (*Igfbp3*), insulin (*Ins*), Ins receptor (*Insr*) and insulin receptor substrate 2 (*Irs2*). At 5 d*pp*, we found an increased expression of *Igf1r* in IUGR rats compared to controls (*P* = 0.05). *Irs2* expression was also upregulated in 20 d*pp* IUGR rats (*P* < 0.05) as well as the expression of *Insr* at 40 d*pp* (*P* < 0.05).

### Testis size

**Figure 2:**
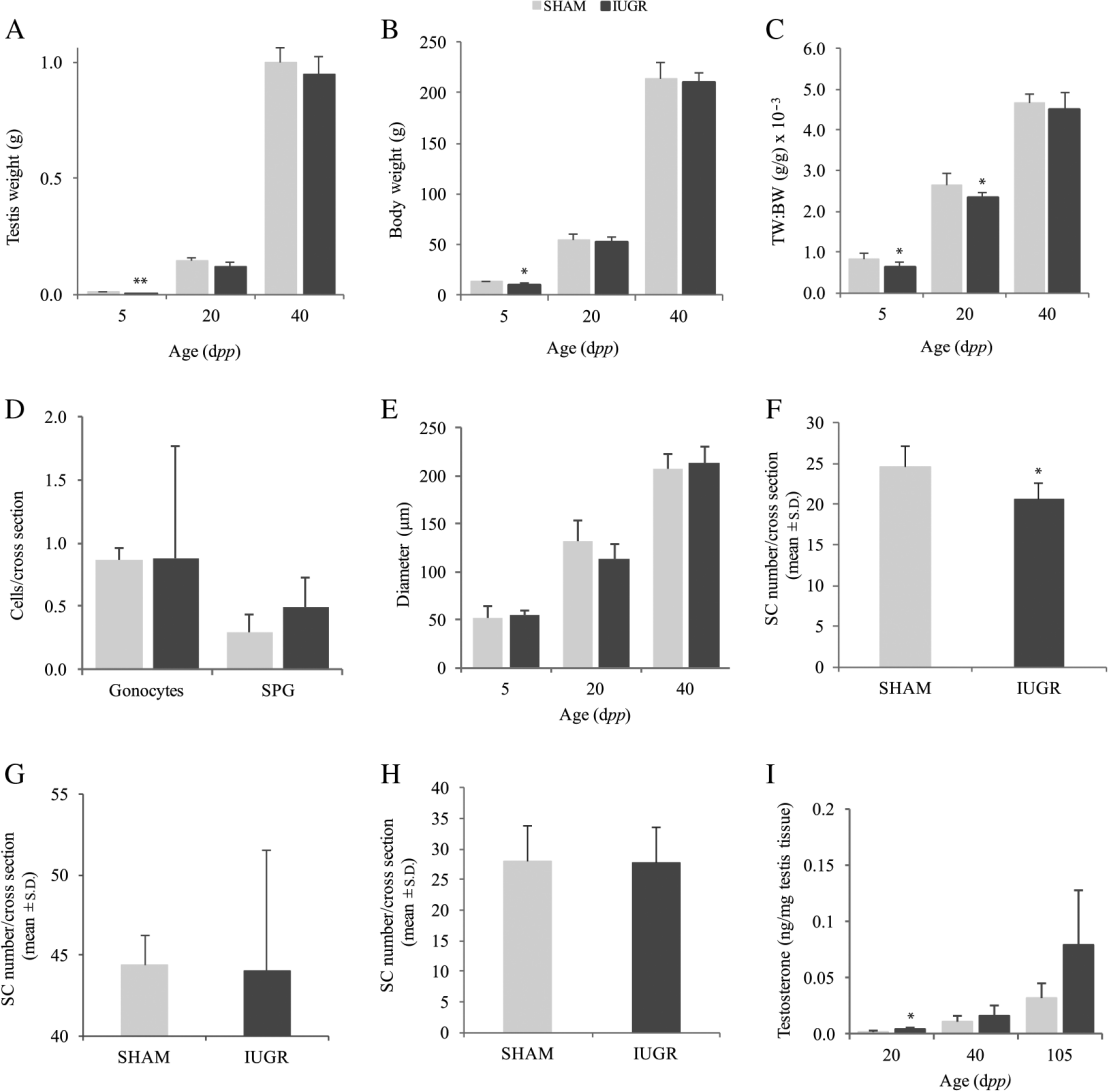
IUGR rats have reduced testis weight at 5 and 20 d*pp*. (A) Mean ± s.d. of testis weight, (B) mean body weight and (C) mean testis weight to body weight ratio ×10^−3^ in IUGR and *sham* rats at 5, 20 and 40 d*pp*. (D) Gonocyte (G) and spermatogonia (SPG) counts in 5 d*pp* rats were not different between IUGR and *shams*. Mean number ± s.d. of G and SPG per tubule cross-section. (E) Tubule diameters were not different between IUGR and *sham* rats. Mean diameter ± s.d. at 5, 20 and 40 d*pp*. Sertoli cell number was reduced in IUGR rats at 5 d*pp*. Mean number ± s.d. of Sertoli cell per tubule cross section in (F) 5 d*pp*, (G) 20 d*pp* and (H) 40 d*pp* old IUGR and *sham* animals. (I) Intratesticular testosterone was increased in IUGR rats at 20 d*pp*. Mean ± s.d. of intratesticular testosterone concentrations at 20 and 40 d*pp* rats (ng/mg testis tissue ×10^−3^). Asterisks denote a significant difference compared to *shams*; *P < 0.05, **P < 0.001.

Testes from IUGR rats were significantly reduced in weight at 5 d*pp* compared to *sham* rats (5.9 ± 1.2 vs 10.8 ± 1.9 mg, *P* < 0.001) (Fig. 2A) as well as the mean body weight (9.6 ± 1.9 vs 13.2 ± 0.6 g, *P* < 0.05) (Fig. 2B). The mean of both testes weight and body weight of IUGR rats caught-up from 20 d*pp* (Fig. 2A and B).

When testes weight was normalized to body weight, the ratio of testes weights to body weight was significantly reduced at 5 d*pp* and 20 d*pp* in IUGR compared to *sham* rats (*P* < 0.05). At 40 d*pp*, the difference was no longer significant although a tendency to reduction remained in IUGR rats compared to *shams* (Fig. 2C).

### Testis histology

Histological examination of testicular cross-sections at 5, 20 and 40 d*pp* by PAS staining revealed no change of the morphology between IUGR and *sham* animals (Supplementary Fig. 1).

The migration of gonocytes (G) from the center of the seminiferous cords to the basement membrane is necessary for their differentiation into spermatogonia (SPG) and survival (Orth *et al*. 1997). To explore whether the postnatal migratory pathway of GC was altered by IUGR, we assessed the number of G and SPG per seminiferous tubule cross-section in 5 d*pp* rats. GCs attached to the basement membrane of seminiferous tubules were considered SPG and GC in the tubule lumen were considered G. Moreover, SPG and G were distinguished based on their distinctive morphology, with gonocytes being large round cells with prominent nuclei, and spermatogonia being semicircular-shaped cells with the flattest edge attached to the basement membrane (Manku & Culty 2015). The number of seminiferous tubule cross-sections (T) per image was also counted such that the number of cells per seminiferous tubular cross-section (G/T or SPG/T) could be calculated. Due to variation of tubules that could be counted, 24.4 ± 9.8 tubules/animal were analyzed in the IUGR group and 19.8 ± 5.3 tubules/animal in the *sham* one. The mean number of G/T was not different between IUGR and *sham* rats at 5 d*pp* (0.89 ± 0.3 vs 0.86 ± 0.1) as there was no difference in the mean number of SPG/T (0.49 ± 0.24 vs 0.29 ± 0.13) (Fig. 2D).

For the estimation of seminiferous tubule diameters, cross-sections of 20 seminiferous tubules *per* animal were randomly chosen and measured in each animal at 5, 20 and 40 d*pp*. Three measurements of each cross-section of seminiferous tubules were made using ImageJ software and the average between them was calculated.

IUGR rats did not exhibit a significant reduction in the diameter of seminiferous tubules compared to controls at any age (Fig. 2E).

For quantification of the SC population, WT1-positive cells were counted in 38.0 ± 5.40 tubules/animal in the IUGR group and in 33.9 ± 10.9 tubules/animal in the *sham* group.

SC number was significantly lower in IUGR rats at 5 d*pp* compared to controls (*P* < 0.05) (Fig. 2F), whereas it was not significantly different between the two groups at 20 and 40 d*pp* (Fig. 2G and H).

### Intratesticular and serum hormonal measurements

To investigate the influence of IUGR on LC function, intratesticular testosterone concentration (ITT) was measured at 20 and 40 d*pp*.

ITT was significantly increased in IUGR at 20 d*pp* compared to *shams* (3.7 ± 1.4 vs 2.1 ± 0.9 pg/mg of testis tissue, *P* < 0.05), whereas it was not different at 40 d*pp*, even though a tendency to increased levels in IUGR rats was noted (Fig. 2I and Supplementary Table 3).

Intratesticular estradiol levels at 20 and 40 d*pp* were assessed to verify the integrity of testicular aromatase activity after IUGR. No significant difference was found in the intraparenchymal estradiol concentrations at these ages (data not shown).

To assess whether the observed difference in ITT levels was dependent on hypothalamic–pituitary regulation, LH and FSH serum levels were measured at 20 and 40 d*pp*. No significant differences in gonado­trophins serum levels were observed between IUGR and *sham* rats (Supplementary Table 3).

## Discussion

Prenatal events causing modifications in the gonads can have long-term consequences on reproductive health and gamete integrity. In this regard, epidemiological studies have reported conflicting results on testicular function in IUGR individuals, whereas animal studies have shown that IUGR induced by maternal undernutrition can impair testicular structure, functionality and sperm parameters. Animal models manipulating uteroplacental blood flow to induce IUGR are more appropriate to investigate IUGR morbidity in relation to Western countries, where malnutrition is seldom the cause of IUGR. We used a UPI rat model to investigate the effects of IUGR on gene expression, structure and functionality in the postnatal testis.

### Cell metabolism

Comparison of the gene expression profile between IUGR and *shams* showed changes in genes involved in the regulation of metabolic processes. Fetal hypoxia increased the expression of the mitochondrial enzymes *Cs* (the first enzyme in the citric acid cycle) and *Hadh* (an indicator of fatty acids β-oxidation) in 5 d*pp* rat testicular tissue, suggesting a modification of the metabolic balance within testicular cells to cope with the reduced supply from ligated uterine arteries.

Increased *Slc2a1* (also known as *Glut-1*) expression (the main glucose transporter across the plasma membrane) was detected by Lane and coworkers in the liver of newborn IUGR rats and normalized after the neonatal period, suggesting a transient adaptive response to the limited blood provision during the fetal period (Lane *et al*. 1999).

Interestingly, we detected a modification of *Glut-1* expression only in the testis of young adult rats (40 d*pp*). SCs sustain developing germ cells providing them with metabolic substrates. To do this, SCs internalize glucose from the interstitial space via GLUT-1 and GLUT-3 glucose transporters (Galardo *et al*. 2008) and use it to produce lactate via glycolysis pathway. Lactate produced from SCs is the main energy source for spermatogenic cells and a survival factor (Erkkila *et al*. 2002, Boussouar & Benahmed 2004). Therefore, the upregulation of *Glut-1* observed in peripubertal IUGR rats may be necessary to support germ cell metabolism at onset of spermatogenesis, when metabolic requirements rise considerably, suggesting that compensatory mechanisms can be modulated according to the testicular developmental stage. Most importantly, these findings open the hypothesis that a dysregulation of GLUT-1 determining a blunted glucose uptake from SCs could play a role in male idiopathic subfertility. This issue merits further investigation and if a role of GLUT-1 in male infertility should emerge, targeting GLUT-1 could open a therapeutic option for these patients.

### SC function and testis size

Several genes selected for their functional role in SCs were dysregulated in response to UPI; at 5 d*pp*, *Sox9* expression was decreased in IUGR rats compared to *shams*. *Sox9* is a transcription factor of the SOX (SRY-related HMG box) family, which has a primary role in mammalian testis differentiation (Kanai *et al*. 2005). From testicular cord formation, *Sox9* is selectively expressed in SCs and has therefore been proposed as a SC marker (Morais da Silva *et al*. 1996, Hemendinger *et al*. 2002). Accordingly, SC number was reduced at this age, suggesting that UPI can interfere with the attainment of an appropriate number of SCs at birth. However, among several SC-specific transcripts analyzed, only *Sox9* was affected in IUGR animals at this age, indicating that the transcriptional response is not only a reflection of the reduced SC number. The functional consequences of these transcriptional changes remain to be studied.

In line with the finding of reduced SC number, the major determinant of gonadal size in the prepubertal age (Rey 2003), testis weight normalized to body weight was significantly lower in IUGR rats at 5 and 20 d*pp*, indicating that IUGR affected gonadal weight to a greater extent than whole body weight. Genovese and coworkers reported a significant reduction of testis weight and SC number in adult rats whose growth was restricted both *in utero* and postnatally by reduction of the nutritional intake. However, the ratio of testis weights to body weights was increased, indicating that general body development was more severely affected than the testis development itself (Genovese *et al*. 2010).

Despite a reduction of seminiferous tubule diameter is expected to occur when SC number reduces, we did not observe such a difference. This discrepancy has been observed by others (Hazra *et al*. 2014) and remains to be explained. However, the magnitude of SC reduction in our study, although significant, was of ~16% compared to controls, probably being not sufficient to produce a reduction in the tubular diameter.

To our knowledge, our report is the first demonstrating that IUGR can produce postnatal changes in the testis size, which appear to be mediated by a reduction in SC number.

Catch-up growth in both testis and body weight occurred by 20 d*pp*, and testis weight normalized to body weight was comparable between groups at 40 d*pp*.

In 20 and 40 d*pp* old rats, the gene changes detected in IUGR vs *sham* animals may be interpreted in the light of testis weight catch-up growth. In postnatal life, immature SCs proliferate until 16 d*pp* in the rat. After this period, intratesticular testosterone rise induces SC maturation and AMH production is downregulated (Sharpe *et al*. 2003). The increased *Amh* expression observed in 20 d*pp* IUGR animals suggests a delay in SC maturation that would allow a prolonged mitotic phase and the production of more SCs to repopulate the testis and ultimately restore testis size. In support of this hypothesis, *Cdkn1a* expression was downregulated in 20 d*pp* IUGR rats in comparison to *shams*. *Cdkn1a* is a cell cycle inhibitor that regulates adult SC number and whose knockout has been shown to produce an increase in SC number and in testis weight (Holsberger *et al*. 2005). Similarly, the expression of *Fshr* was increased in IUGR rats at 20 and 40 d*pp* compared to *shams*, suggesting an amplification of FSH signaling. FSH is the main factor that regulates SC function and allows an adequate SC proliferation (Petersen & Soder 2006). Although the proliferation rate of SCs was not assessed in this study, these transcriptional changes in IUGR animals can hypothetically act in concert to restore SC number and ultimately testicular size.

Finally, in 40-d*pp*-old IUGR rats, we observed a downregulation of *Kitl*, encoding for the stem cell factor (SCF), produced exclusively by SCs and whose interaction with the receptor c-kit expressed in SPG is crucial for spermatogenesis (Petersen & Soder 2006). Thus, implications of an altered SCF/c-kit signaling on GC development in the IUGR rat testis cannot be excluded.

### LC function

Data from different animal species subjected to malnutrition during pregnancy have shown reduced serum testosterone levels in male offspring (Zambrano *et al*. 2005, Toledo *et al*. 2011). In contrast to the effects observed in malnutrition models, two studies focusing on renal effects of UPI in rats, coincidentally reported increased serum levels of testosterone in adult rats (Ojeda *et al*. 2007, Baserga *et al*. 2009). As serum testosterone levels exhibit circadian fluctuations, we measured intraparenchymal testosterone levels, a more reliable measure of testosterone production by the testis. In line with the aforementioned studies, we detected significantly increased intratesticular testosterone levels in 20 d*pp* IUGR rats compared to *shams*. At 40 d*pp*, testosterone was 1.4-fold increased, compared to the control group, although it did not reach statistical significance. Serum gonadotrophins levels were not different between IUGR and *sham* rats at any age, indicating that the increased testosterone production was independent from pituitary regulation. Nevertheless, two markers of LC function, *Cyp17* and *Tspo* were upregulated in 5 d*pp* IUGR rats compared to *shams*. As both enzymes are involved in androgen synthesis, these changes could be preliminary to the significant increase in ITT levels observed at 20 d*pp*, when gene expression was, however, no longer different between the two groups.

The increased testosterone levels may be finalized at providing an equal amount of circulating testosterone despite the reduced testis size. Moreover, androgens indirectly mediate SC proliferation and maturation (Johnston *et al*. 2004) and have been shown to contribute to the restoration of testis weight in hypogonadal mice more substantially than FSH alone (Haywood *et al*. 2003). Thus, the increased intratesticular testosterone concentrations in IUGR rats may also contribute at normalizing SC number and allowing catch-up growth of testis size.

### IGF/insulin pathways

IGF/insulin pathways have been shown to play an important role in testis, being involved in the regulation of both SC and LC function. Selective knockout of *Igf1r* and *Insr* in mice SCs decreases the proliferation rate of immature SCs during the early postnatal period, ultimately affecting adult testis size and daily sperm output (Pitetti *et al*. 2013). Moreover, *in vitro* and *in vivo* studies in rodents demonstrated a determinant role of IGF-I in regulating survival and testosterone production by LC in postnatal life (Baker *et al*. 1996, Colon *et al*. 2007, Svechnikov *et al*. 2010). Our expression analysis revealed an upregulation of three different key components of the IGF/insulin pathway (*Igf1r*, *Irs2* and *Insr*) in IUGR rats compared to *shams* at 5, 20 and 40 d*pp*, respectively, suggesting a possible influence of this system both on SC number and testosterone production by LC during the catch-up growth phase occurring in IUGR rat testis from 20 d*pp*.

## Conclusion

Collectively, these results provide evidence that IUGR induced by UPI interfere with testis development, as indicated by testicular size impairment and reduced SC number in neonatal rats. The recovery of testicular weight observed by three weeks of age appears to be regulated at multiple levels, by a fine and complex interplay between genes involved in SC function, steroidogenesis and cellular homeostasis, which ultimately produce a restoration of the organ size. Further studies are needed to corroborate these findings and to verify whether the resumption of SC number and testis weight reflects an intact organ function in terms of reproductive performance and gamete integrity.

Moreover, perturbation of SC development has been proposed as a player in the pathogenesis of the testicular dysgenesis syndrome, a complex of different diseases (including low sperm count and increased risk of testicular cancer) that may share the same causal mechanisms and has been tightly related to IUGR in epidemiological studies. The IUGR-induced alterations in gene expression profile of SCs observed in our study could represent a link between these conditions and need to be taken into account to orientate future investigations.

Although preliminary, our study sheds light on previously unknown consequences of an event commonly affecting human gestations. For the obvious reason of limited access to human tissues, genetic arrangement and morphology of the human testis after IUGR is largely unknown. This work could serve as reference to orientate future studies and translate these observations into human pathology.

## Supplementary data

This is linked to the online version of the paper at http://dx.doi.org/10.1530/JOE-16-0418.

## Declaration of interest

The authors declare that there is no conflict of interest that could be perceived as prejudicing the impartiality of the research reported.

## Funding

This study was supported by research grants from the Swedish Research Council, Children’s Cancer Found, Stockholm County Council Research Funds, the Frimurare Foundation and Karolinska Institutet. Valentina Pampanini received grant support from the Sällskapet Barnavård foundation.

## References

[bib1] Arends NJ Boonstra VH Duivenvoorden HJ Hofman PL Cutfield WS Hokken-Koelega AC 2005 Reduced insulin sensitivity and the presence of cardiovascular risk factors in short prepubertal children born small for gestational age (SGA). Clinical Endocrinology 62 44–50. (10.1111/j.1365-2265.2004.02171.x) 15638869

[bib2] Baker J Hardy MP Zhou J Bondy C Lupu F Bellve AR Efstratiadis A 1996 Effects of an Igf1 gene null mutation on mouse reproduction. Molecular Endocrinology 10 903–918. (10.1210/me.10.7.903) 8813730

[bib3] Barker DJ 1990 The fetal and infant origins of adult disease. British Medical Journal 301 1111 (10.1136/bmj.301.6761.1111) 2252919 PMC1664286

[bib4] Barker DJ 1995 Fetal origins of coronary heart disease. British Medical Journal 311 171–174. (10.1136/bmj.311.6998.171) 7613432 PMC2550226

[bib5] Barker DJ Bull AR Osmond C Simmonds SJ 1990 Fetal and placental size and risk of hypertension in adult life. British Medical Journal 301 259–262. (10.1136/bmj.301.6746.259) 2390618 PMC1663477

[bib6] Barker DJ Martyn CN Osmond C Hales CN Fall CH 1993 Growth in utero and serum cholesterol concentrations in adult life. British Medical Journal 307 1524–1527. (10.1136/bmj.307.6918.1524) 8274921 PMC1679540

[bib7] Baserga M Bares AL Hale MA Callaway CW McKnight RA Lane PH Lane RH 2009 Uteroplacental insufficiency affects kidney VEGF expression in a model of IUGR with compensatory glomerular hypertrophy and hypertension. Early Human Development 85 361–367. (10.1016/j.earlhumdev.2008.12.015) 19188030 PMC4447306

[bib8] Boussouar F Benahmed M 2004 Lactate and energy metabolism in male germ cells. Trends in Endocrinology and Metabolism 15 345–350. (10.1016/j.tem.2004.07.003) 15350607

[bib9] Colon E Zaman F Axelson M Larsson O Carlsson-Skwirut C Svechnikov KV Soder O 2007 Insulin-like growth factor-I is an important antiapoptotic factor for rat leydig cells during postnatal development. Endocrinology 148 128–139. (10.1210/en.2006-0835) 17023532

[bib10] Dupont C Cordier AG Junien C Mandon-Pepin B Levy R Chavatte-Palmer P 2012 Maternal environment and the reproductive function of the offspring. Theriogenology 78 1405–1414. (10.1016/j.theriogenology.2012.06.016) 22925651

[bib11] Engelbregt MJ Houdijk ME Popp-Snijders C Delemarre-van de Waal HA 2000 The effects of intra-uterine growth retardation and postnatal undernutrition on onset of puberty in male and female rats. Pediatric Research 48 803–807. (10.1203/00006450-200012000-00017) 11102550

[bib12] Engelbregt MJ van Weissenbruch MM Popp-Snijders C Delemarre-van de Waal HA 2002 Delayed first cycle in intrauterine growth-retarded and postnatally undernourished female rats: follicular growth and ovulation after stimulation with pregnant mare serum gonadotropin at first cycle. Journal of Endocrinology 173 297–304. (10.1677/joe.0.1730297) 12010637

[bib13] Erkkila K Aito H Aalto K Pentikainen V Dunkel L 2002 Lactate inhibits germ cell apoptosis in the human testis. Molecular Human Reproduction 8 109–117. (10.1093/molehr/8.2.109) 11818513

[bib14] Forsen T Eriksson JG Tuomilehto J Osmond C Barker DJ 1999 Growth in utero and during childhood among women who develop coronary heart disease: longitudinal study. British Medical Journal 319 1403–1407. (10.1136/bmj.319.7222.1403) 10574856 PMC28284

[bib15] Gagnon R 2003 Placental insufficiency and its consequences. European Journal of Obstetrics and Gynecology and Reproductive Biology 110 (Supplement 1) S99–S107. (10.1016/S0301-2115(03)00179-9) 12965097

[bib16] Galardo MN Riera MF Pellizzari EH Chemes HE Venara MC Cigorraga SB Meroni SB 2008 Regulation of expression of Sertoli cell glucose transporters 1 and 3 by FSH, IL1 beta, and bFGF at two different time-points in pubertal development. Cell and Tissue Research 334 295–304. (10.1007/s00441-008-0656-y) 18802725

[bib17] Genovese P Nunez ME Pombo C Bielli A 2010 Undernutrition during foetal and post-natal life affects testicular structure and reduces the number of Sertoli cells in the adult rat. Reproduction in Domestic Animals 45 233–236. (10.1111/j.1439-0531.2008.01244.x) 19281598

[bib18] Gnessi L Fabbri A Spera G 1997 Gonadal peptides as mediators of development and functional control of the testis: an integrated system with hormones and local environment. Endocrine Reviews 18 541–609. (10.1210/er.18.4.541) 9267764

[bib19] Hales CN Barker DJ 2001 The thrifty phenotype hypothesis. British Medical Bulletin 60 5–20. (10.1093/bmb/60.1.5) 11809615

[bib20] Hales CN Barker DJ Clark PM Cox LJ Fall C Osmond C Winter PD 1991 Fetal and infant growth and impaired glucose tolerance at age 64. British Medical Journal 303 1019–1022. (10.1136/bmj.303.6809.1019) 1954451 PMC1671766

[bib21] Haywood M Spaliviero J Jimemez M King NJ Handelsman DJ Allan CM 2003 Sertoli and germ cell development in hypogonadal (hpg) mice expressing transgenic follicle-stimulating hormone alone or in combination with testosterone. Endocrinology 144 509–517. (10.1210/en.2002-220710) 12538611

[bib22] Hazra R Upton D Jimenez M Desai R Handelsman DJ Allan CM 2014 In vivo actions of the Sertoli cell glucocorticoid receptor. Endocrinology 155 1120–1130. (10.1210/en.2013-1940) 24424066

[bib23] Hemendinger RA Gores P Blacksten L Harley V Halberstadt C 2002 Identification of a specific Sertoli cell marker, Sox9, for use in transplantation. Cell Transplantation 11 499–505. 12428738

[bib24] Hernandez MI Mericq V 2008 Impact of being born small for gestational age on onset and progression of puberty. Best Practice and Research Clinical Endocrinology and Metabolism 22 463–476. (10.1016/j.beem.2008.02.003) 18538286

[bib25] Hokken-Koelega AC 2002 Timing of puberty and fetal growth. Best Practice and Research Clinical Endocrinology and Metabolism 16 65–71. (10.1053/beem.2002.0181) 11987899

[bib26] Holsberger DR Buchold GM Leal MC Kiesewetter SE O’Brien DA Hess RA Franca LR Kiyokawa H Cooke PS 2005 Cell-cycle inhibitors p27Kip1 and p21Cip1 regulate murine Sertoli cell proliferation. Biology of Reproduction 72 1429–1436. (10.1095/biolreprod.105.040386) 15728790

[bib27] Jensen RB Vielwerth S Larsen T Greisen G Veldhuis J Juul A 2007 Pituitary-gonadal function in adolescent males born appropriate or small for gestational age with or without intrauterine growth restriction. Journal of Clinical Endocrinology and Metabolism 92 1353–1357. (10.1210/jc.2006-2348) 17227800

[bib28] Johnston H Baker PJ Abel M Charlton HM Jackson G Fleming L Kumar TR O’Shaughnessy PJ 2004 Regulation of Sertoli cell number and activity by follicle-stimulating hormone and androgen during postnatal development in the mouse. Endocrinology 145 318–329. (10.1210/en.2003-1055) 14551232

[bib29] Kanai Y Hiramatsu R Matoba S Kidokoro T 2005 From SRY to SOX9: mammalian testis differentiation. Journal of Biochemistry 138 13–19. (10.1093/jb/mvbib98) 16046443

[bib30] Kotsampasi B Balaskas C Papadomichelakis G Chadio SE 2009 Reduced Sertoli cell number and altered pituitary responsiveness in male lambs undernourished in utero. Animal Reproduction Science 114 135–147. (10.1016/j.anireprosci.2008.08.017) 18814977

[bib31] Lane RH Crawford SE Flozak AS Simmons RA 1999 Localization and quantification of glucose transporters in liver of growth-retarded fetal and neonatal rats. American Journal of Physiology 276 E135–E142. 9886959 10.1152/ajpendo.1999.276.1.E135

[bib32] Law CM Barker DJ Osmond C Fall CH Simmonds SJ 1992 Early growth and abdominal fatness in adult life. Journal of Epidemiology and Community Health 46 184–186. (10.1136/jech.46.3.184) 1645067 PMC1059546

[bib33] Main KM Jensen RB Asklund C Hoi-Hansen CE Skakkebaek NE 2006 Low birth weight and male reproductive function. Hormone Research 65 (Supplement 3) 116–122. (10.1159/000091516) 16612124

[bib34] Manku G Culty M 2015 Mammalian gonocyte and spermatogonia differentiation: recent advances and remaining challenges. Reproduction 149 R139–R157. (10.1530/REP-14-0431) 25670871

[bib35] Morais da Silva S Hacker A Harley V Goodfellow P Swain A Lovell-Badge R 1996 Sox9 expression during gonadal development implies a conserved role for the gene in testis differentiation in mammals and birds. Nature Genetics 14 62–68. (10.1038/ng0996-62) 8782821

[bib36] Ojeda NB Grigore D Yanes LL Iliescu R Robertson EB Zhang H Alexander BT 2007 Testosterone contributes to marked elevations in mean arterial pressure in adult male intrauterine growth restricted offspring. American Journal of Physiology: Regulatory, Integrative and Comparative Physiology 292 R758–R763. (10.1152/ajpregu.00311.2006) 16917022

[bib37] Orth JM Qiu J Jester WF Jr Pilder S 1997 Expression of the c-kit gene is critical for migration of neonatal rat gonocytes in vitro. Biology of Reproduction 57 676–683. (10.1095/biolreprod57.3.676) 9283007

[bib38] Persson I Ahlsson F Ewald U Tuvemo T Qingyuan M von Rosen D Proos L 1999 Influence of perinatal factors on the onset of puberty in boys and girls: implications for interpretation of link with risk of long term diseases. American Journal of Epidemiology 150 747–755. (10.1093/oxfordjournals.aje.a010077) 10512428

[bib39] Petersen C Soder O 2006 The sertoli cell – a hormonal target and ‘super’ nurse for germ cells that determines testicular size. Hormone Research 66 153–161. (10.1159/000094142) 16804315

[bib40] Peterside IE Selak MA Simmons RA 2003 Impaired oxidative phosphorylation in hepatic mitochondria in growth-retarded rats. American Journal of Physiology: Endocrinology and Metabolism 285 E1258–E1266. (10.1152/ajpendo.00437.2002) 14607783

[bib41] Pitetti JL Calvel P Zimmermann C Conne B Papaioannou MD Aubry F Cederroth CR Urner F Fumel B Crausaz M 2013 An essential role for insulin and IGF1 receptors in regulating sertoli cell proliferation, testis size, and FSH action in mice. Molecular Endocrinology 27 814–827. (10.1210/me.2012-1258) 23518924 PMC5416760

[bib42] Puglianiello A Germani D Antignani S Tomba GS Cianfarani S 2007 Changes in the expression of hypothalamic lipid sensing genes in rat model of intrauterine growth retardation (IUGR). Pediatric Research 61 433–437. (10.1203/pdr.0b013e3180332d4e) 17515867

[bib43] Rae MT Rhind SM Fowler PA Miller DW Kyle CE Brooks AN 2002 Effect of maternal undernutrition on fetal testicular steroidogenesis during the CNS androgen-responsive period in male sheep fetuses. Reproduction 124 33–39. (10.1530/rep.0.1240033) 12090916

[bib44] Rey R 2003 Regulation of spermatogenesis. Endocrine Development 5 38–55. (10.1159/000069300) 12629891

[bib45] Selak MA Storey BT Peterside I Simmons RA 2003 Impaired oxidative phosphorylation in skeletal muscle of intrauterine growth-retarded rats. American Journal of Physiology: Endocrinology and Metabolism 285 E130–E137. (10.1152/ajpendo.00322.2002) 12637257

[bib46] Sharpe RM McKinnell C Kivlin C Fisher JS 2003 Proliferation and functional maturation of Sertoli cells, and their relevance to disorders of testis function in adulthood. Reproduction 125 769–784. (10.1530/rep.0.1250769) 12773099

[bib47] Silveira PP Portella AK Goldani MZ Barbieri MA 2007 Developmental origins of health and disease (DOHaD). Jornal de Pediatria 83 494–504. (10.2223/jped.1728) 18074050

[bib48] Skakkebaek NE Rajpert-De Meyts E Main KM 2001 Testicular dysgenesis syndrome: an increasingly common developmental disorder with environmental aspects. Human Reproduction 16 972–978. (10.1093/humrep/16.5.972) 11331648

[bib49] Sofikitis N Giotitsas N Tsounapi P Baltogiannis D Giannakis D Pardalidis N 2008 Hormonal regulation of spermatogenesis and spermiogenesis. Journal of Steroid Biochemistry and Molecular Biology 109 323–330. (10.1016/j.jsbmb.2008.03.004) 18400489

[bib50] Svechnikov K Landreh L Weisser J Izzo G Colon E Svechnikova I Soder O 2010 Origin, development and regulation of human Leydig cells. Hormone Research in Paediatrics 73 93–101. (10.1159/000277141) 20190545

[bib51] Thompson LP Al-Hasan Y 2012 Impact of oxidative stress in fetal programming. Journal of Pregnancy 2012 582748 (10.1155/2012/582748) 22848830 PMC3403156

[bib52] Toledo FC Perobelli JE Pedrosa FP Anselmo-Franci JA Kempinas WD 2011 In utero protein restriction causes growth delay and alters sperm parameters in adult male rats. Reproductive Biology and Endocrinology 9 94 (10.1186/1477-7827-9-94) 21702915 PMC3141647

[bib53] Tsirka AE Gruetzmacher EM Kelley DE Ritov VH Devaskar SU Lane RH 2001 Myocardial gene expression of glucose transporter 1 and glucose transporter 4 in response to uteroplacental insufficiency in the rat. Journal of Endocrinology 169 373–380. (10.1677/joe.0.1690373) 11312153

[bib54] Veening MA van Weissenbruch MM Roord JJ de Delemarre-van Waal HA 2004 Pubertal development in children born small for gestational age. Journal of Pediatric Endocrinology and Metabolism 17 1497–1505. (10.1515/jpem.2004.17.11.1497) 15570986

[bib55] Vuguin PM 2007 Animal models for small for gestational age and fetal programming of adult disease. Hormone Research 68 113–123. (10.1159/000100545) PMC4287248 17351325

[bib56] Vuguin P Raab E Liu B Barzilai N Simmons R 2004 Hepatic insulin resistance precedes the development of diabetes in a model of intrauterine growth retardation. Diabetes 53 2617–2622. (10.2337/diabetes.53.10.2617) 15448092

[bib57] Wigglesworth JS 1964 Experimental growth retardation in the foetal rat. Journal of Pathology and Bacteriology 88 1–13. 14194979

[bib58] Zambrano E Rodriguez-Gonzalez GL Guzman C Garcia-Becerra R Boeck L Diaz L Menjivar M Larrea F Nathanielsz PW 2005 A maternal low protein diet during pregnancy and lactation in the rat impairs male reproductive development. Journal of Physiology 563 275–284. (10.1113/jphysiol.2004.078543) 15611025 PMC1665555

[bib59] Zambrano E Guzman C Rodriguez-Gonzalez GL Durand-Carbajal M Nathanielsz PW 2014 Fetal programming of sexual development and reproductive function. Molecular and Cellular Endocrinology 382 538–549. (10.1016/j.mce.2013.09.008) 24045010

